# Hybrid Bacterial Foraging and Particle Swarm Optimization for detecting Bundle Branch Block

**DOI:** 10.1186/s40064-015-1240-z

**Published:** 2015-09-04

**Authors:** Padmavathi Kora, Sri Ramakrishna Kalva

**Affiliations:** Department of ECE, GRIET, Bachupally, Hyderabad, 500090 India; Department of ECE, Velagapudi Ramakrishna Siddhartha Engineering College, Kanuru, Vijayawada, India

**Keywords:** Bundle branch block, BFO, PSO, BFPSO, LM NN classifier

## Abstract

Abnormal cardiac beat identification is a key process in the detection of heart diseases. Our present study describes a procedure for the detection of left and right bundle branch block (LBBB and RBBB) Electrocardiogram (ECG) patterns. The electrical impulses that control the cardiac beat face difficulty in moving inside the heart. This problem is termed as bundle branch block (BBB). BBB makes it harder for the heart to pump blood effectively through the heart circulatory system. ECG feature extraction is a key process in detecting heart ailments. Our present study comes up with a hybrid method combining two heuristic optimization methods: Bacterial Forging Optimization (BFO) and Particle Swarm Optimization (PSO) for the feature selection of ECG signals. One of the major controlling forces of BFO algorithm is the chemotactic movement of a bacterium that models a test solution. The chemotaxis process of the BFO depends on random search directions which may lead to a delay in achieving the global optimum solution. The hybrid technique: Bacterial Forging–Particle Swarm Optimization (BFPSO) incorporates the concepts from BFO and PSO and it creates individuals in a new generation. This BFPSO method performs local search through the chemotactic movement of BFO and the global search over the entire search domain is accomplished by a PSO operator. The BFPSO feature values are given as the input for the Levenberg–Marquardt Neural Network classifier.

## Background

Heart diseases are the most important cause of human mortality globally. Every year, 9.4 million deaths are attributed to heart diseases. This includes 51 % of deaths due to strokes and 45 % of deaths due to coronary heart diseases. Most of the cardiac diseases are caused due to the risk factors such as unhealthy diet, high blood pressure, tobacco usage, obesity, diabetes and physical inactivity.

Bundle branch block (BBB) is a type heart abnormality (arrhythmia) that causes death in adults. BBB is developed when there is a block along the path of electrical pulses within the heart. A condition in which there is a delay in the heart conduction system in the lower chambers and can be observed through the changes in the Electrocardiogram (ECG). ECG is a cost effective tool for analyzing cardiac abnormalities. The diagnostic accuracy of BBB depends on the precise detection of ECG features. ECG changes in Left bundle branch block (LBBB) are:

- Increased QRS complex duration ( >0.12 s).
- Increased Q wave amplitude.
- Abnormal T wave.

ECG changes in right bundle branch block (RBBB) are:

- Increased QRS complex duration ( >0.12 s).
- RSR’ format.
- T wave inversion.

**Fig. 1:**
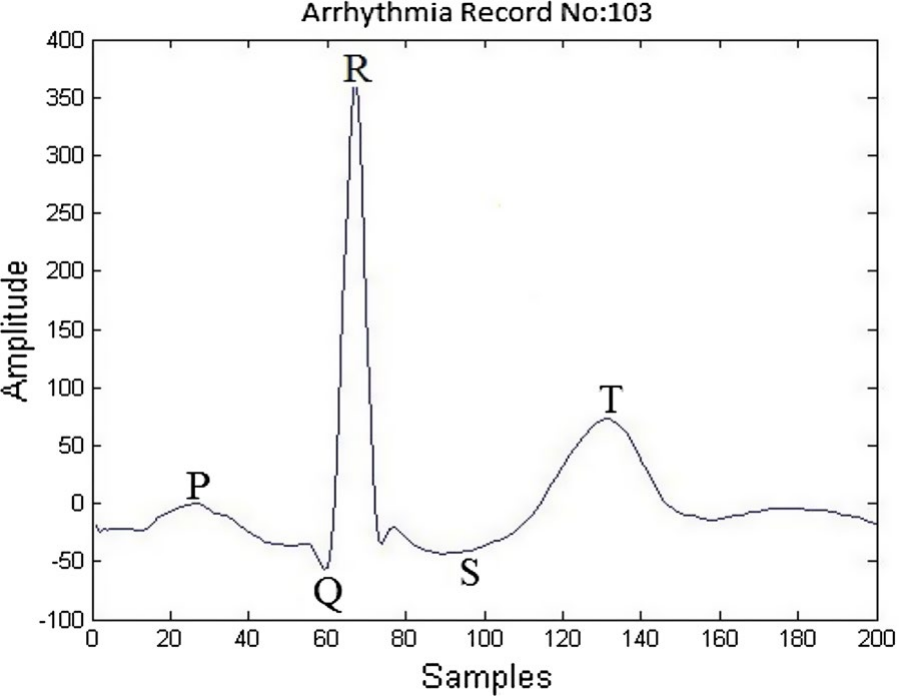
Normal beat

**Fig. 2:**
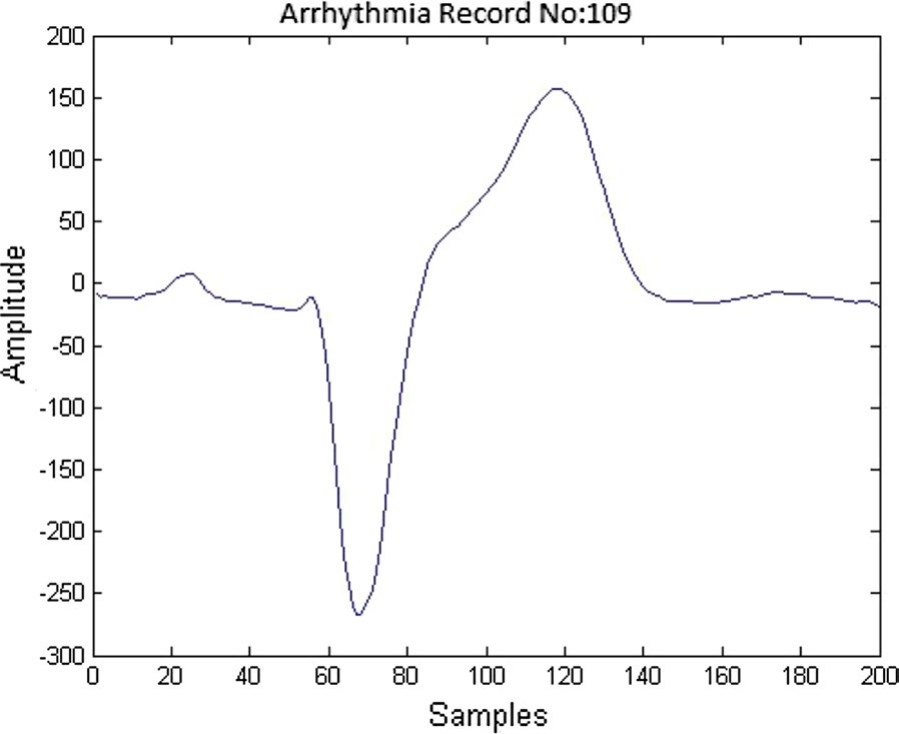
Left bundle branch block

**Fig. 3:**
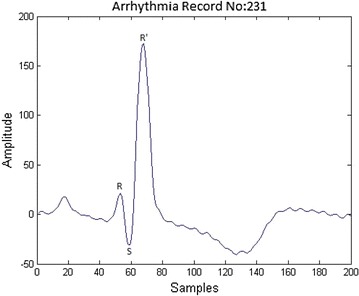
Right bundle branch block

The waveform changes in the different types of ECG beats have been shown in the Figs. 1, 2 and 3.

The diagnoses of BBB with the help of ECG consists of three main stages: preprocessing, feature extraction and classification. The first step in preprocessing focuses on the noise removal using filters. The second stage in preprocessing is segmentation which separates ECG files into beats. The samples that are extracted from each beat contains non uniform samples. The non uniform samples in each beat are converted into uniform samples of size 200 by using a technique called re-sampling. The re-sampled ECG beat is shown in Fig. 1. The next stage in BBB is the feature extraction. In this paper, Bacterial Foraging–Particle Swarm Optimization (BFPSO) technique is used as a feature extraction (optimization) method. It belongs to the family of swarm based optimization techniques. Swarm based algorithms have gained increased attention of the scientists and engineers in solving several engineering problems which can not be solved by traditional gradient based methods. The feature selection of ECG is very difficult to arrive at statistically. A large number of swarm based methods have been used in order to solve a few engineering problems like Genetic Algorithm (GA) Back (1996), Particle Swarm Optimization (PSO) Kennedy and Eberhart (1995), Bacterial Foraging Optimization (BFO) (Passino 2002; Liu and Passino 2002; Ahmad et al. 2014) etc.

BFO has poor convergence behavior over the other naturally inspired optimization algorithms because it (BFO) follows the local search through a random search process (chemotaxtic). Its overall performance depends more on the growth of search space dimensionality. BFO has very few successful engineering applications in optimal control engineering (Mishra and Bhende 2007), harmonic estimation theory (Mishra 2005), transmission path loss reduction (Tripathy et al. 2006; Tripathy and Mishra 2007), machine learning optimization (Cho et al. 2007) and so on.

PSO (Eberhart and Kennedy 1995) is a swarm based optimization algorithm and it takes inspiration from a group of birds or a group of fish etc. It has an advantage of high convergence speed as it finds the global optimum point using gbest, pbest values. However, the disadvantage of PSO is that, it gets trapped into the local minimum.

In this paper, a novel hybrid optimization method concurrently combines the BFO (Tang et al. 2006) with the PSO. The proposed hybrid algorithm fulfills the local search by using Chemotactic operation of BFO whereas the global search is accomplished by a PSO operator. Using this combination, it maintains a balance between ‘exploration’ and ‘exploitation’ and enjoys the best of both the algorithms, BFO and PSO (Abd-Elazim and Ali 2013). The proposed BFPSO method has been used in order to solve a few engineering problems (Dasgupta et al. 2009; Datta and Misra 2008; Biswas et al. 2007). BFPSO has been compared with the normal GA, PSO and BFO. The following comparative measures has been used to study the (1) Accuracy of the final solution (2) Convergence speed. Such comparison shows the superiority of the proposed algorithm over the traditional methods. This algorithm outperforms both PSO and BFO over a few ECG benchmark data sets for the classification of ECG.

**Fig. 4:**
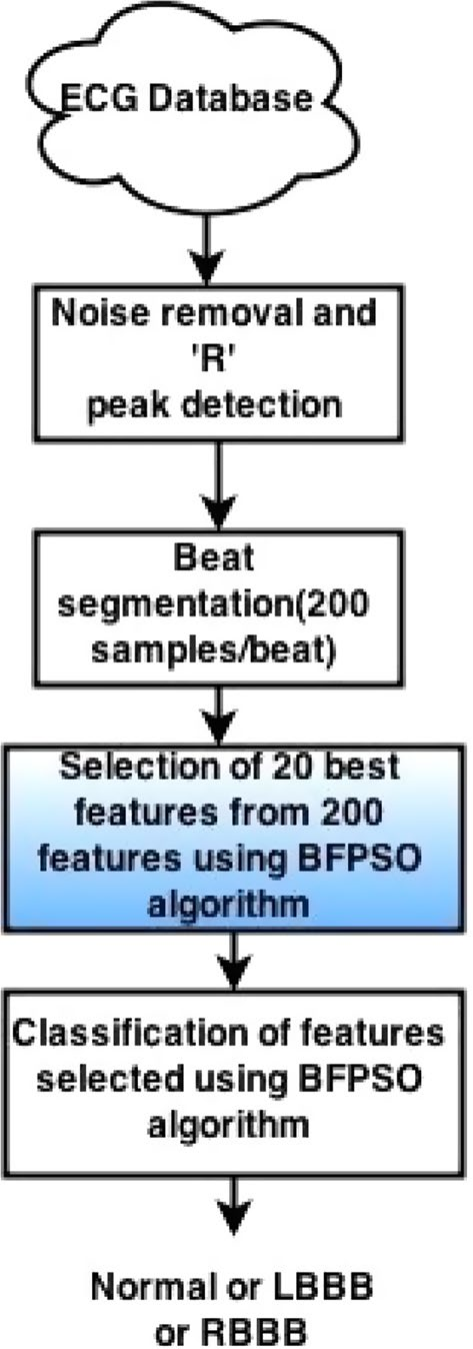
ECG classification flow diagram

The ECG classification flow diagram is shown in the Fig. 4.

## Pre processing

### Data collection

**Table 1:** MIT-BIH record numbers

Record	NB	LBBB	RBBB
100	2237	0	0
101	1858	0	0
103	2080	0	0
106	1505	0	0
109	0	2490	0
111	0	2121	0
118	0	0	2164
123	1513	0	0
124	0	0	1529
207	0	1457	85

To prove the performance of BFPSO, the usual MIT BIH arrhythmia database (Goldberger et al. 2000) is considered. The data used in this algorithm confines to 11 recordings that consists of 5 normal, 3 LBBB and 3 RBBB for a duration of 60 min at 360 Hz sampling rate. The file numbers of 11 recordings for normal and BBB are shown in Table 1.

### Noise removal

**Fig. 5:**
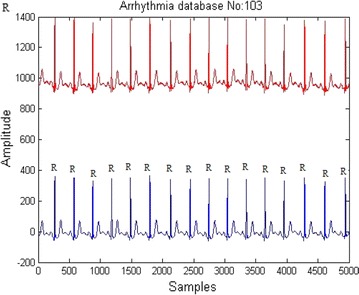
ECG Baseline Wander removal. *Up signal* original signal. *Down signal* baseline Wander removed signal

Sgolay FIR filter was used to remove the baseline wander present in the signal as shown Fig. 5.

### R peak detection and beat segmentation of ECG

**Fig. 6:**
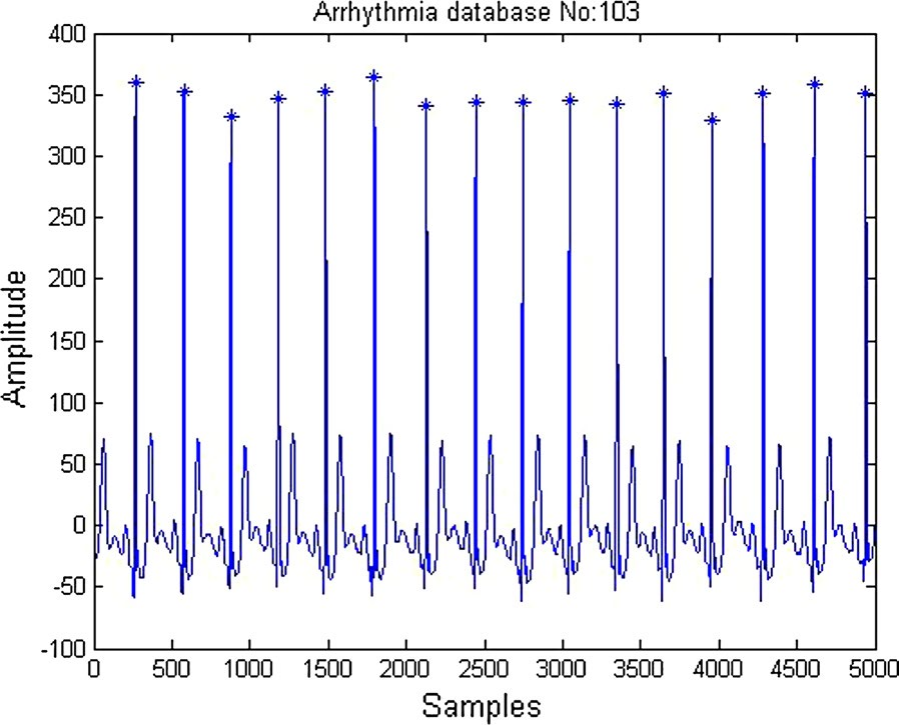
ECG R peak detection

**Fig. 7:**
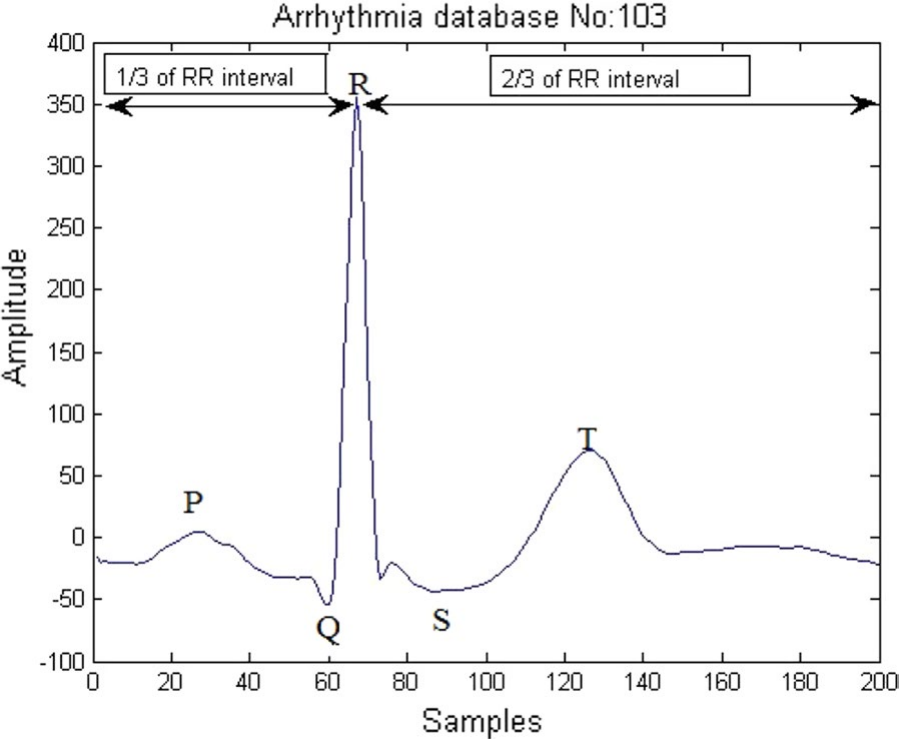
ECG beat segmentation


Distance between two R peaks is called RR interval as depicted in Fig. 6. 2/3 rds of the RR interval samples to the right of R peak and 1/3 rds of the RR interval samples to the left of R peak were considered as one beat as in Fig. 7. Each beat after segmentation was re-sampled to 200 samples.

1/3 of RR interval:(R peak):2/3 of RR interval.

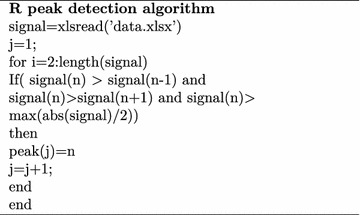


## Feature extraction of ECG signal

### Genetic algorithm (GA)

Genetic algorithm (Goldberg and David 1989) simulates the process of evolution in nature. The methods of GA can be applied to solve many real-world problems. Genetic algorithms allow each successive generation of solutions to evolve from the previous generation’s strengths. GA is an adaptive heuristic method for searching the global optimum point. It maps the search space into a genetic space. That is, every possible key is encoded with a vector called chromosome. Each element of the chromosome represents a gene. All the chromosomes make up a population. The strength of the population is estimated according to the fitness function. The fitness function is used to measure the fitness of each chromosome.

Initial population in GA is randomly created. The first step in GA is to evaluate the fitness of the each candidate in the population. Then GA is applied for minimization or maximization of the fitness. GA then uses three steps to produce the next generation from the current generation. They are

- Reproduction.
- Crossover.
- Mutation.

*Reproduction* Reproduction is based on the Darwinian theory of “Survival of the fittest”. GA eliminates the population of low fitness and keeps the population of high fitness. This whole process is repeated, and the population of high fitness move to the next generation until a good population (individuals) is found.

*Cross over* In cross over process, two parents are selected from good individuals and are used to produce a new offspring. The process of crossover continues for a fixed number of iterations or until a termination condition is satisfied.

*Mutation* It introduces new features in the offspring that is completely different from their forerunners. Hence mutation inherently introduces genetic diversity in the present population.

The main objective of genetic feature selection stage is to reduce the dimensionality (Raymer et al. 2000) of the problem before the supervised neural network classification process. GA, which solves the optimization problems using the methods of evolution, has proved to be a promising one. GA evaluates each individual’s fitness as well as the quality of the solution. The fittest individuals are more eligible to enter the next generation as a population. After a required number of generations, the final set of optimal population with the fittest chromosomes will emerge. The process of selection, crossover and mutation continues for a fixed number of generations or till a termination condition is satisfied. Genetic algorithms have been used for selecting the optimal subspace in which the projected data gives higher recognition accuracy.

### Particle Swarm Optimization (PSO)

PSO (Melgani and Bazi 2008) is a kind of swarm based optimization method developed by Eberhart and Kennedy inspired from the behavior of a flock of birds. Each particle in the group flies in the search domain with a velocity and it tries to attain the best velocity according to its own previous best (pbest) and its companions’ best (gbest) flying experience.

**Fig. 8:**
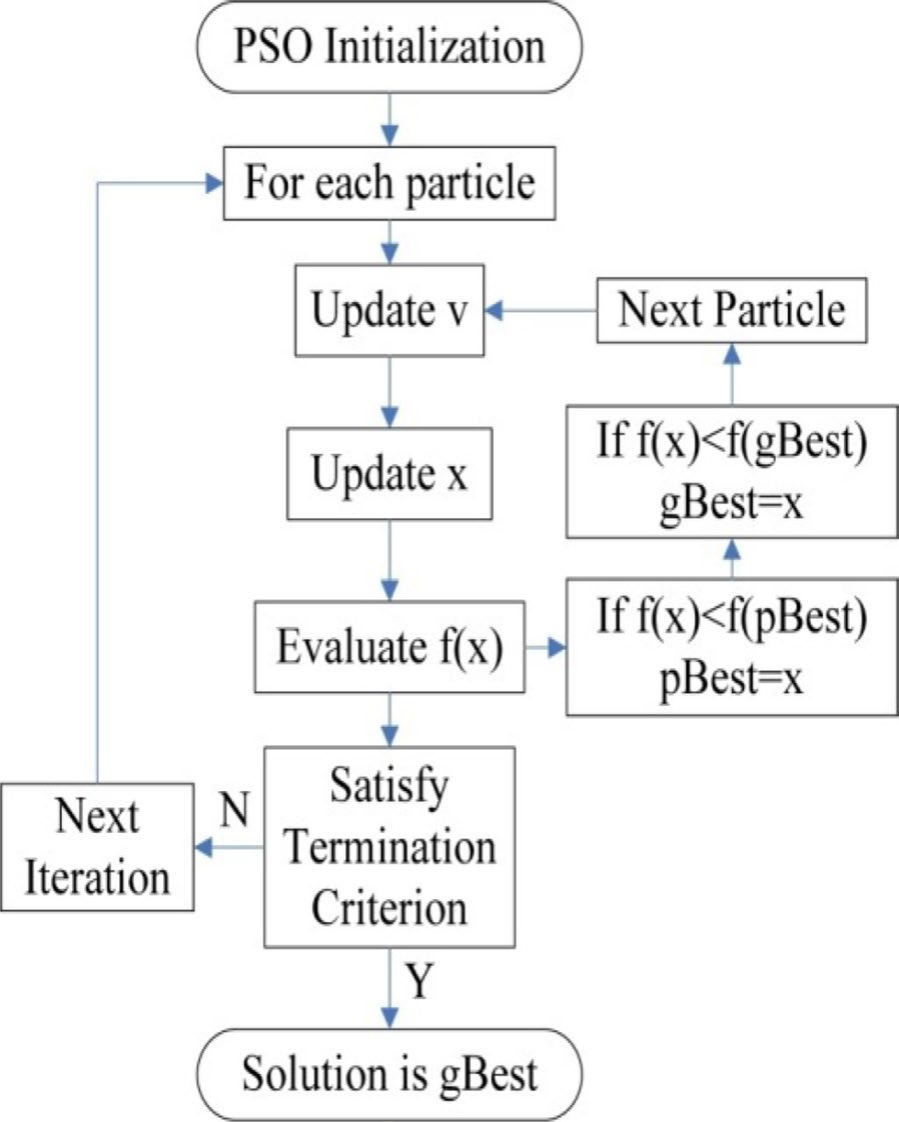
PSO flowchart

Each particle in the search space tries to adjust its position (location) using the following measures 
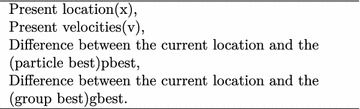


The basic idea behind PSO Eberhart and Kennedy (1995) is, accelerating each particle in search space towards the gbest and the pbest values, at each step with the random acceleration as shown in Fig. 8.

The advantage of using PSO over other optimization techniques is its simplicity. And very few parameters need to be adjusted. Due to this, PSO has been widely used in a variety of applications. In an n-dimensional search space, $X_{i}$ = (x1, x2, x3,...,xn), let the particles be initialized with positions $X_{i}$ and velocities $V_{i}$ and the fitness is calculated based on particle positional coordinates as the input values. Then the particles are moved into new positions using the equations below:

1 $$\begin{aligned} V_{i}(i+1) = \omega .V_{i}(i) + C1.\phi 1.(Pbest -X_{i}(i)) + C2.\phi 2. (gbest-X_{i}(i)) \end{aligned}$$

2 $$\begin{aligned} X_{i}(i+1) = X_{i}(i) + V_{i}(i+1) \end{aligned}$$

### Bacterial Foraging Optimization (BFO)

In the year 2002, BFO was developed by the researcher Passino (2002), Liu and Passino (2002) which relies on a selection procedure that get rid of the bacterium with poor search methods. Several generations with poor foraging methods are eliminated whereas only the organisms with good search strategy survive signifying the “survival of the fittest”.

Bacterial foraging activity of “*E. coli*” bacteria (Tang et al. 2006) is used as the inspiration for extracting (optimizing) the features of ECG. Feature selection may be a international optimization problem in machine learning, that optimize/reduce the number of features, redundant and noisy features, removes unsuitable features, this leads to acceptable accuracy.

Bacteria move in a random manner to find increasing nutrients. Hence, this optimization technique is useful when gradient of cost function is not known. BFO is good because of it’s less mathematical complexity. The BFO is a non-gradient optimization technique. It mimics the search mechanism of *E. coli* bacterium. *E. coli* tries to maximize its food intake per unit time spent in search. The three operating steps of each bacterium per unit area are

- Chemotaxis
- Swarming
- Reproduction

- Chemotaxis: The random walk of *E. coli* bacterium can be explained in two steps
  1. Swimming.
  2. Tumbling.
    Basically the *E. coli* bacteria will move in two alternative ways. It will swim for a specific amount of time in one direction then it is going to tumble (change direction). It will alternate between these two modes of operation for its entire life period. Say x(i) represents *i*th bacteria and C, the step size taken within the random direction specified by the swim length. In the process of chemotaxis the x(i+1) of the bacteria could also be given by
    3 $$\begin{aligned} x(i+1)=x(i)+C(i)\frac{Del(i)}{Del(i)Del^{T}(i)} \end{aligned}$$
    where ‘$Del$ ’ is a random vector $\epsilon$ (−1,1). The simulated chemotactic movement of an *E. coli* bacterium may be viewed as a random hill climbing.

(b) *Swarming* In *E. coli* bacteria swarm behavior is observed like in several other species, where the complex and stable spacio-temporal groups are formed in a semi-solid nutrient medium. The *E. coli* forms themselves like a traveling ring and moving down towards the nutrient food. The *E. coli* bacteria releases an attractant, aspartate when its cells are excited by a high level of succinate. This helps them to arrange into groups and thus they move as coaxial patterns of groups with high density. The cell-cell signaling in* E. coli* swarm may be calculated by the following Rosenbrock function.
  4 $$\begin{aligned} \sum _{i=1}^{d-1}[100(x_{i+1}-x_{i}^2)^2+(x_{i}-1)^2] \end{aligned}$$
  where d is the dimension and x(i) is the *i*th bacterium.

(c) *Reproduction* The unhealthy bacteria finally die while the remaining healthier bacteria (those giving higher value of the cost value) asexually split into two bacteria, and they are placed in their respective positions. So the total size of the bacteria swarm remains constant.

**Fig. 9:**
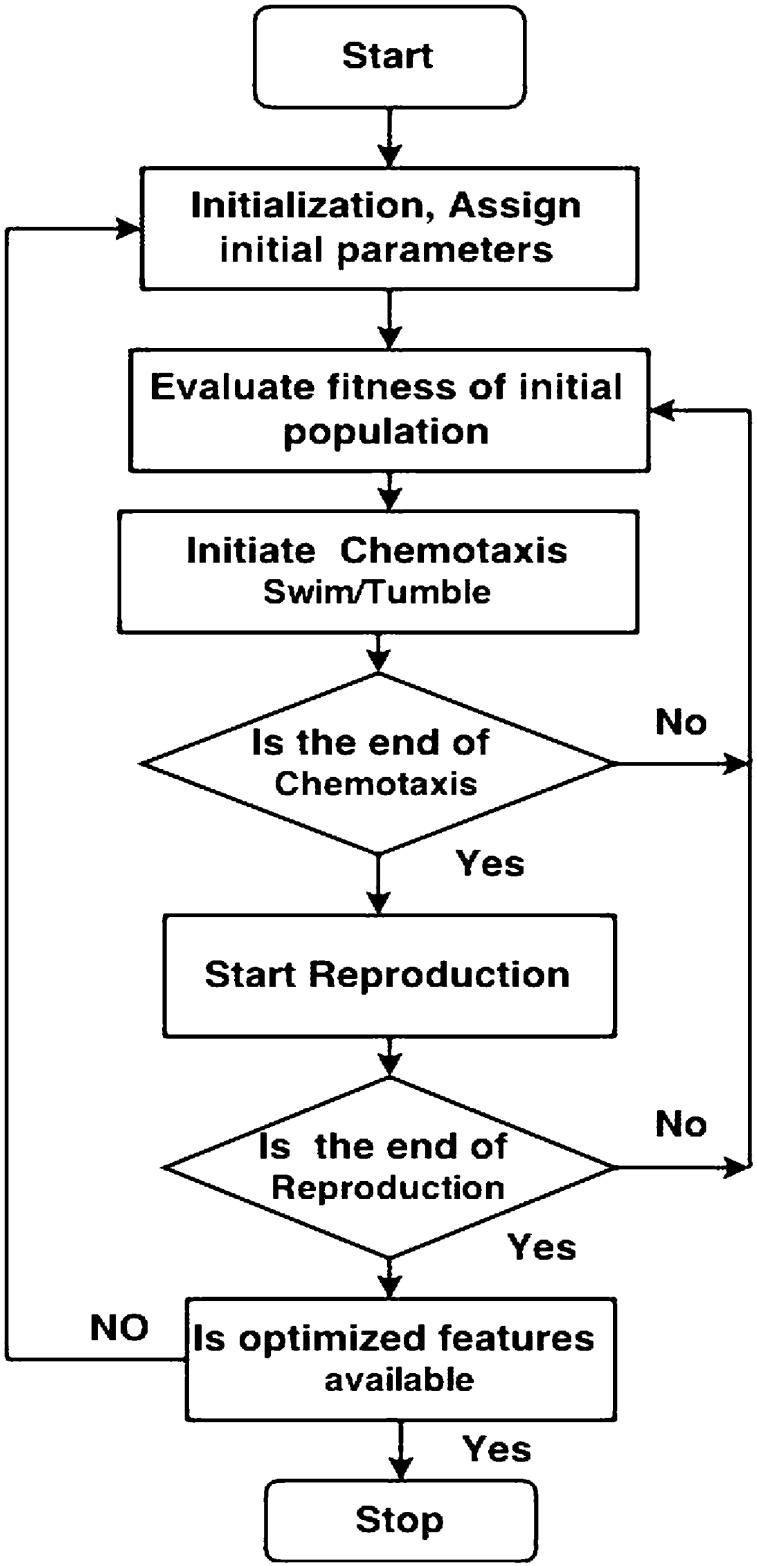
BFO flow chart


The complete pseudo code and flowchart for feature optimization using BFO is given below Fig. 9.

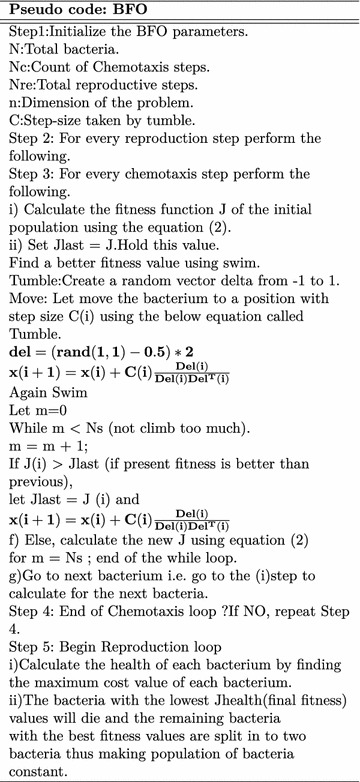


### Bacterial Foraging Particle Swarm Optimization algorithm (BFPSO)

This section introduces a hybrid technique consisting of BFO and PSO algorithms. The two basic steps involved in the development of the proposed algorithm are:

1. Global search through the PSO operator followed by
2. Local search through the BFO (chemotaxis) which fine tunes the solution.

Advantages of this combination are

1. Algorithm is not be trapped into the local minimum.
2. Convergence speed will be increased.

In this hybrid combination, PSO performs a global search and produces a near optimal solution very rapidly which is then followed by a local search by BFO which fine-tunes the solution and gives an optimum solution of high accuracy. PSO has an inherent disadvantage of being trapped in the local optimum but has high convergence speed whereas BFO has the drawback of having a very poor convergence speed but has the ability of not being trapped in the local optima. 
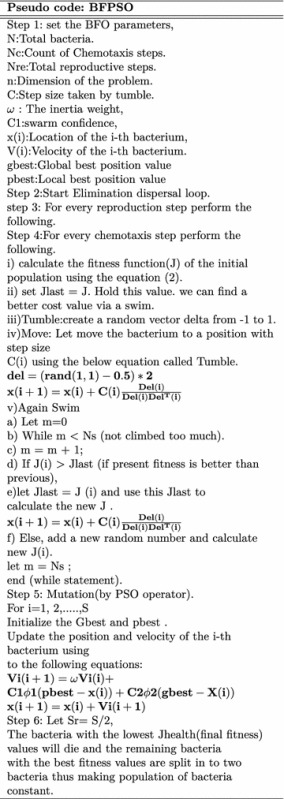


**Fig. 10:**
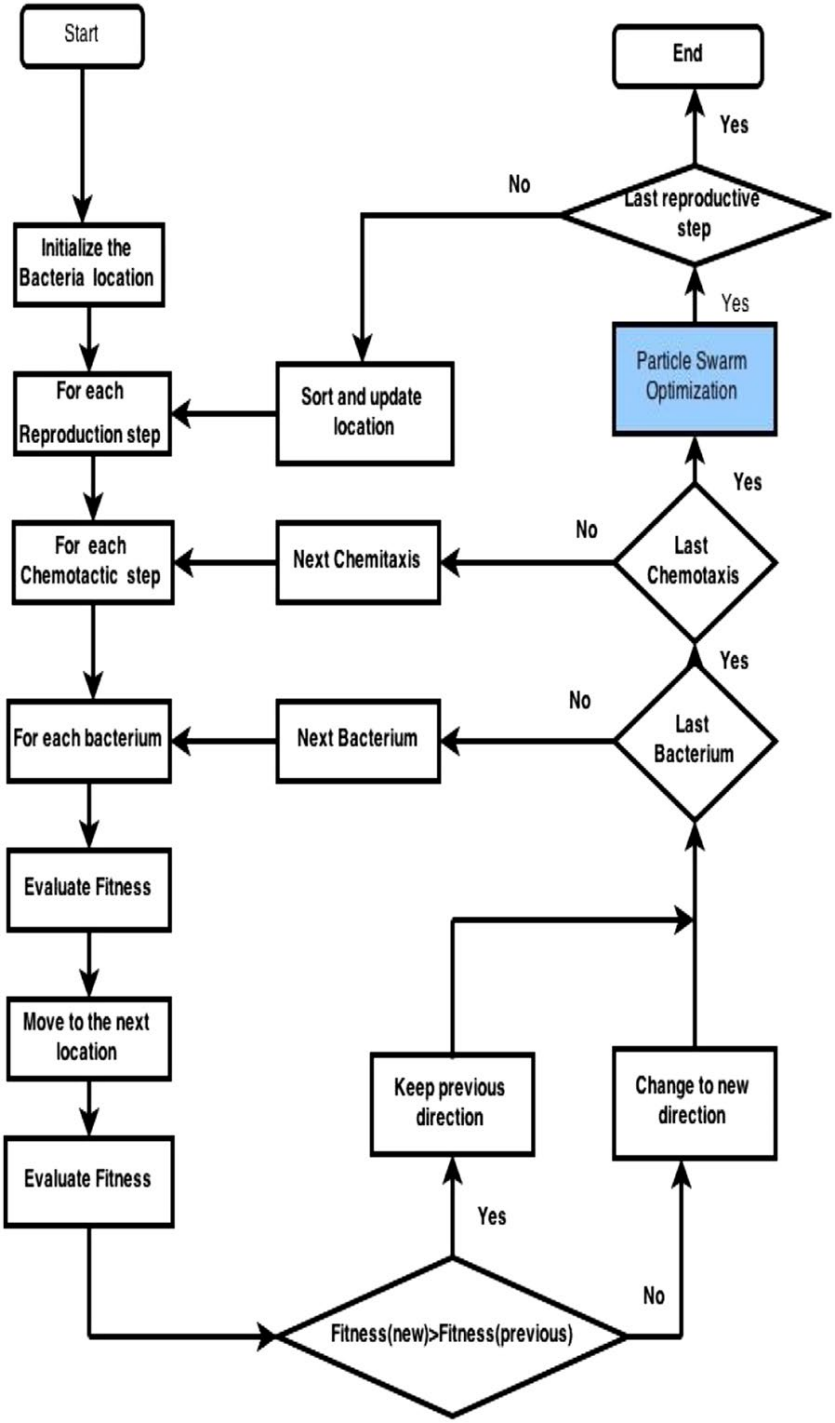
BFPSO flow chart

After a specified number of complete swims, the resulting solution is stored in the descending order. A detailed description of the complete algorithm can be traced in Fig. 10.

In the present approach, after undergoing a chemotactic step, each bacterium also gets mutated by a PSO operator. In this step, each bacterium is stochastically attracted towards the gbest position and the local search in different regions is taken care of by the BFO (chemotaxis step) algorithm.

The main objective of BFPSO feature selection stage is to reduce the features of the problem before the supervised neural network classification. In all the wrapper algorithms used, BFPSO solves optimization problems using the methods of evolution and has emerged as a promising one.

## Classification of BFPSO features

The extracted features (20 features) from BFPSO algorithm are classified using different types of classification techniques such as KNN, SVM, Neural Network classifiers.

### Levenberg–Marquardt neural network (LM NN)

In this work for the detection of BBB, back propagation Levenberg–Marquardt neural network (LMNN) (Ibn Ibrahimy et al. 2013) is used. This NN provides rapid execution of the network to be trained, which is the main advantage in the neural signal processing applications Sapna et al. (2012).

The NN was designed to work well if it was built with 20 input neurons, 10 neurons in the hidden layer and 3 neurons in the output layer.

The performance of this algorithm is compared with Scalar Conjugate Gradient (SCG) NN. The LMNN algorithm is a robust and a very simple method for approximating a function. SCG NN method provides conjugate directions of search instead of performing a linear search. The network is trained with 1800 ECG beats, and tested with 1006 ECG beats. The total number of iterations are set to 1000 and mean square error less than 0.001. The main advantage of this algorithm is that the time required to train the network is less.

## Results

ECG features before optimization = [1 2 3 .........200];

The optimized ECG features (20 features) using BFPSO algorithm are given below

Optimized features (column numbers) using BFPSO = [67 68 66 69 65 70 71 64 72 63 73 62 74 61 60 75 59 76 58 77];

These reduced features are given as an input to the Neural Network so that its convergence speed and final accuracy can be increased.

The ECG beats after segmentation are re-sampled to 200 samples/beat. Instead of using morphological feature extraction techniques, in this paper BFPSO is used as the feature extraction technique. Using BFPSO ECG beat features are optimized to 20 features. The BFPSO gives optimized features (best features) for the ECG beat classification. The performance of BFPSO is compared with classical GA, PSO, BFO techniques. The BFO, PS0, BFPSO features are classified using SVM, KNN, SCG NN, LM NN as in the Tables 2, 3, 4, 5 and 6.

- Count of Normal beats used for classification: 9193.
- Count of RBBB beats user for classification: 3778.
- Count of LBBB beats user for classification: 6068.
- Total number of beats used for classification: 19,039.
- Count of correctly classified beats: 18,800.
- Total misclassified beats: 239.

For measuring accuracy two parameters, sensitivity and specificity are calculated using the following equations.

5 $$\begin{aligned}Specificity = \frac{{True\_Negative}}{{True\_Negative + False\_Positive}}{\mkern 1mu} \times {\mkern 1mu} 100{\text{ }} \end{aligned}$$

6 $$\begin{aligned} Sensitivity = \frac{{True\_Positive}}{{True\_Positive + False\_Negative}}{\mkern 1mu} \times {\mkern 1mu} 100 \end{aligned}$$

7 $$\begin{aligned} Accuracy = \frac{{TP + TN}}{{TP + TN + FP + FN}}{\mkern 1mu} \times {\mkern 1mu} 100{\text{ }} \end{aligned}$$

- TP(True_Positive) = Count of all the correctly classified Normal beats.
- TN(True_Negative) = Count of all beats the correctly classified Abnormal beats.
- FP(False_Positive) = Count of Normal beats which are classified as Abnormal.
- FN(False_Negative) = Count of Abnormal beats which are classified as Normal.

**Table 2:** Classification with KNN classifier

Classifier	Sensi (%)	Speci (%)	Accuracy (%)
PSO+KNN	52.5	53.2	65.1
GA+KNN	63.5	67.86	64.55
BFO+KNN	53.5	52.2	53.22
BFPSO+KNN	52.35	53.9	52.17

**Table 3:** Classification with SVM classifier

Classifier	Sensi (%)	Speci (%)	Accuracy (%)
PSO+SVM	71.0	73.13	70.12
GA+SVM	87.87	82.85	84.62
BFO+SVM	76.2	75.47	72.13
BFPSO+SVM	75.5	76.9	76.74

**Table 4:** Classification with SCG NN classifier

Classifier	Sensi (%)	Speci (%)	Accuracy (%)
PSO+SCG NN	86.1	85.3	86.0
GA+SCG NN	67.87	82.85	84.62
BFO+SCG NN	88.2	87.2	87.9
BFPSO+SCG NN	84.42	82.28	83.13

**Table 5:** Classification with LM NN classifier

Classifier	Sensi (%)	Speci (%)	Accuracy (%)
BFO+LM NN	93.34.2	92.2	93.9
PSO+LM NN	91.2	89.2	80.9
GA+ LM NN	95.4	96.2	96.5
BFPSO+LM NN	98.97	98.7	98.1

**Table 6:** Overall classification accuracy with BFPSO features

Classifier	LBBB (%)	RBBB (%)	Normal (%)
KNN	55.2	54.2	52.17
SVM	76.1	75.3	76.74
SCG NN	84.42	82.28	83.13
LM NN	98.2	98.15	98.1

**Fig. 11:**
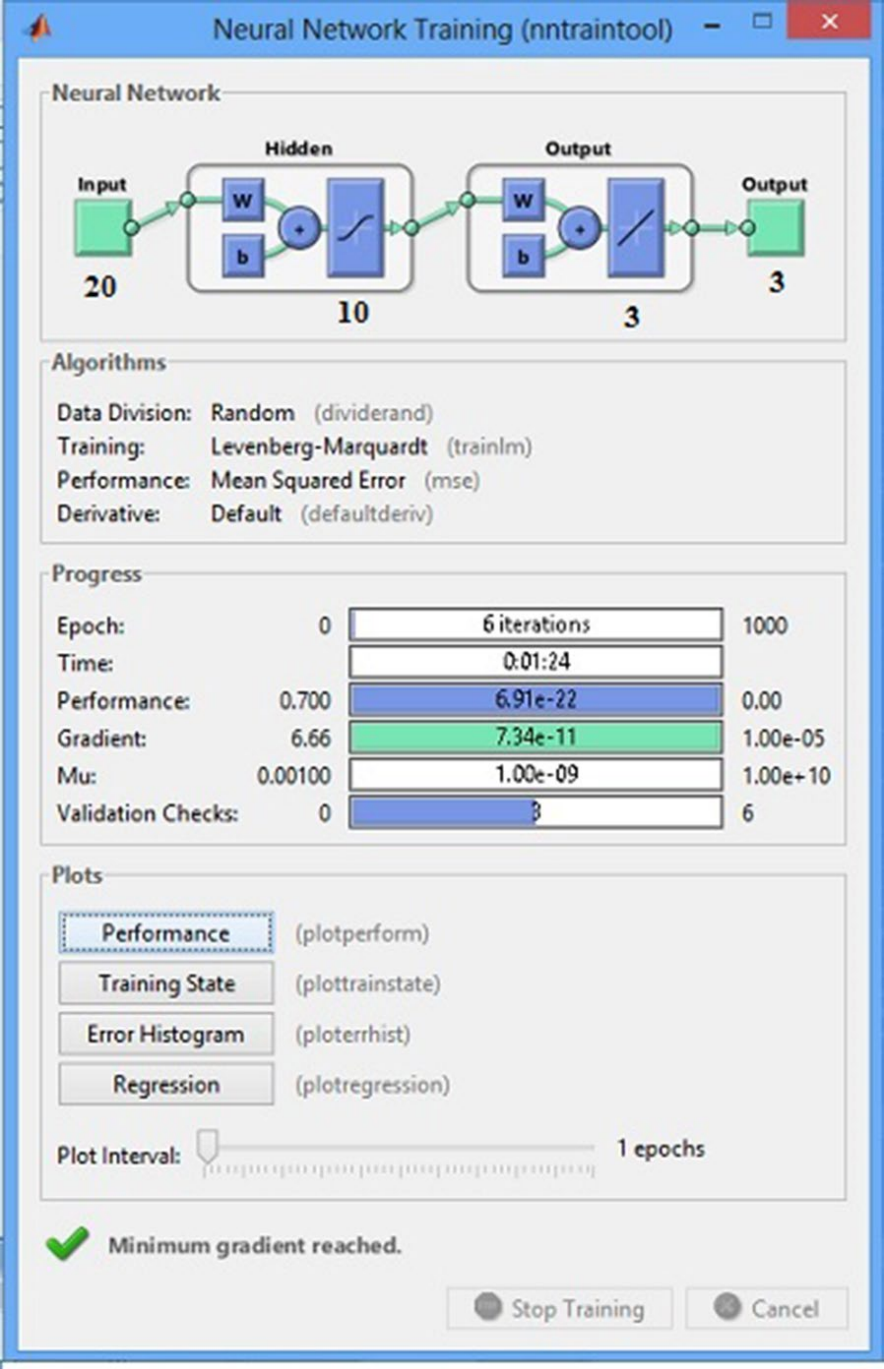
Neural network training with trainlm

In the training mode we applied multilayer NN and checked the network performance and decided if any changes were required to the training process or the data set or the network architecture. First, check the training record, ‘trainlm’ Matlab function as shown in Fig. 11.

**Fig. 12:**
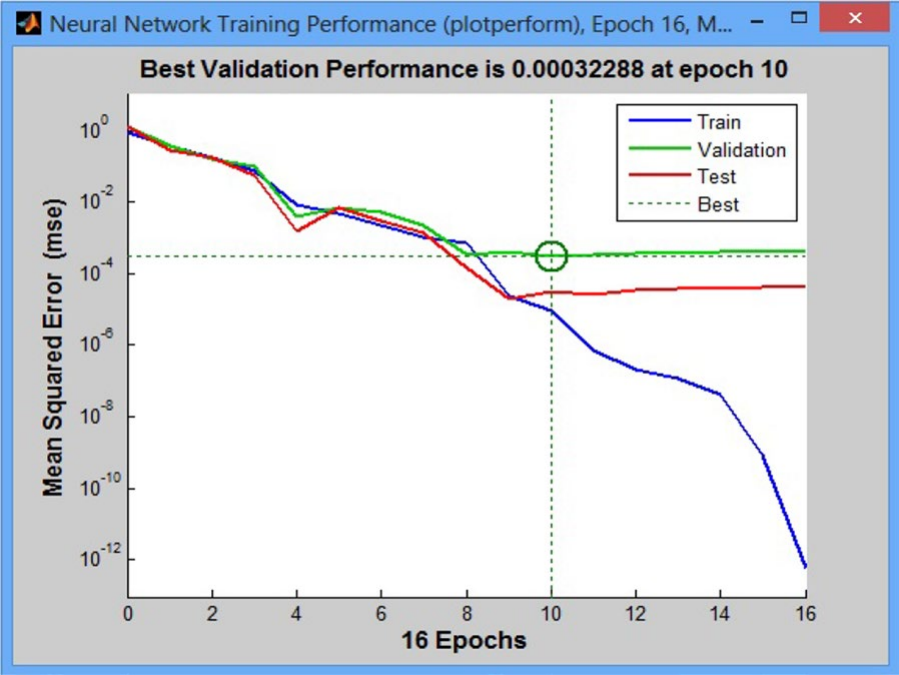
Neural network training performance plot

The property of training indicates that the iteration is up to the point where the performance of the validation has reached a minimum. The training continued for 16 iterations before the stop. The next step is validating the network, a plot of epochs versus Mean Squared Error (MSE), which shows the relationship between the number of epochs of the network to the MSE as shown in Fig. 12. If the training is perfect, the network outputs and the targets have to be exactly equal which is rare in practice.

**Fig. 13:**
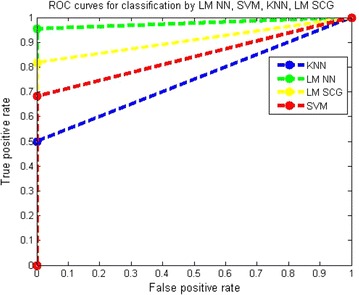
Performance comparison of different classifiers with BFPSO features

Receiver operating characteristic (ROC) curve is plotted for true positive rate (Sensitivity) verses false positive rate (100-Specificity) as in Fig. 13. Each point on the ROC performance curve represents a sensitivity/specificity pair corresponding to a particular parameter. The normal and abnormal classes can be clearly distinguished using the measure of the area under the curve.

## Discussion and conclusion

Early changes of BBB are important because immediate treatment can save the life of the patient from heart failure. There are several methods to detect features of BBB. The RR interval, P wave, statistical methods for feature extraction have some limitations. Accurate detection of features is important for detection of BBB. Nature-inspired algorithms have gained increased attention of scientists and engineers in solving the problems which cannot be solved by the above traditional methods. In our approach, BFPSO features for each ECG beat (BBB, normal) are extracted and the results show that accuracy for the detection of BBB has increased.

In the present study we have developed a simple computational model for the detection of BBB using the BFPSO algorithm. The BFPSO algorithm has been compared with the GA, BFO and PSO. In our study we observed: (1) accuracy, (2) frequency of hitting the optimal point (3) convergence speed. The BFPSO algorithm provides better classification results compared to the original BFO, PSO and GA for all the ECG data. Thus, this optimization method that we have applied may useful for further such investigations.
